# Study on Mechanism of Surfactant Adsorption at Oil–Water Interface and Wettability Alteration on Oil-Wet Rock Surface

**DOI:** 10.3390/molecules30122541

**Published:** 2025-06-10

**Authors:** Xinyu Tang, Yaoyao Tong, Yuhui Zhang, Pujiang Yang, Chuangye Wang, Jinhe Liu

**Affiliations:** College of Chemistry and Chemical Engineering, China University of Petroleum, Qingdao 266580, China; t764236216@163.com (X.T.); tyy13336231519@163.com (Y.T.); 19960026@upc.edu.cn (Y.Z.); 19900020@upc.edu.cn (P.Y.); chwang@upc.edu.cn (C.W.)

**Keywords:** chemical flooding, surfactant, interface properties, heavy oil emulsion

## Abstract

With the depletion of conventional light crude oil reserves in China, the demand for heavy oil exploitation has grown, highlighting the increasing significance of enhanced heavy oil recovery. Surfactants reduce oil–water interfacial tension, modify the wettability of reservoir rocks, and facilitate the emulsification of heavy oil. Consequently, investigating the adsorption behavior of surfactants at oil–water interfaces and the underlying mechanisms of wettability alteration is of considerable importance. In this study, the surface tension of four surfactants and their interfacial tension with Gudao heavy oil were measured. Among these, BS-12 exhibited a critical micelle concentration (CMC) of 6.26 × 10^−4^ mol·dm^−3^, a surface tension of 30.15 mN·m^−1^ at the CMC, and an adsorption efficiency of 4.54. In low-salinity systems, BS-12 achieved an ultralow interfacial tension on the order of 10^−3^ mN·m^−1^, demonstrating excellent surface activity. Therefore, BS-12 was selected as the preferred emulsifier for Gudao heavy oil recovery. Additionally, FT-IR, SEM, and contact angle measurements were used to elucidate the interfacial adsorption mechanism between BS-12 and aged cores. The results indicate that hydrophobic interactions between the hydrophobic groups of BS-12 and the adsorbed crude oil fractions play a key role. Core flooding experiments, simulating the formation of low-viscosity oil-in-water (*O*/*W*) emulsions under reservoir conditions, showed that at low flow rates, crude oil and water interact more effectively within the pores. The extended contact time between heavy oil and the emulsifier led to significant changes in rock wettability, enhanced interfacial activity, improved oil recovery efficiency, and increased oil content in the emulsion. This study analyzes the role of surfactants in interfacial adsorption and the multiphase flow behavior of emulsions, providing a theoretical basis for surfactant-enhanced oil recovery.

## 1. Introduction

As conventional light crude oil reserves decline, heavy oil has emerged as an increasingly important petroleum resource that requires efficient development [1]. At present, thermal recovery methods remain the dominant technique for heavy oil extraction globally. However, due to the inherent limitations of thermal technologies, composite cold production techniques have gained significant attention. These techniques utilize the reservoir native temperature and substantially reduce energy consumption [2,3]. Chemical enhanced oil recovery (EOR) technologies primarily improve displacement efficiency through multiple mechanisms: reducing oil–water interfacial tension, altering rock surface wettability, and promoting oil–water emulsification to decrease the viscosity of heavy oil [4,5,6]. A significant portion of crude oil is trapped in reservoir rock pores by capillary forces [7]. Surfactants effectively reduce the interfacial tension between oil and water [8,9], modify the wettability of reservoir rocks, minimize capillary effects, facilitate heavy oil emulsification, and enhance the ability of water or other displacing fluids to contact and mobilize oil, thereby significantly improving heavy oil recovery.

The wettability of reservoir rock formations is crucial in determining the mobility of heavy oil, as changes in wettability significantly affect the detachment of crude oil from pore surfaces [10]. As an essential parameter in reservoir evaluation [11], wettability influences the microscopic distribution and migration of oil, gas, and water within rock pores and subsequently governs seepage patterns and recovery strategies. Most heavy oil reservoirs initially display water-wet characteristics; however, the adsorption of asphaltenes or other polar components from crude oil can alter the rock surface to an oil-wet state. This transition severely impedes the mobility of heavy oil through porous media, complicating recovery operations. To optimize wettability conditions on rock surfaces, various methods can be employed to modify and regulate wettability [12]. A thorough understanding of the principles governing rock surface wettability is critical for developing effective strategies for wettability modification.

Previous studies have extensively explored the use of various surfactants for modifying reservoir wettability. Jarrahian et al. [13] systematically investigated the mechanisms of wettability alteration in carbonate rocks using multiple experimental approaches. An FTIR analysis of aged and surfactant-treated core samples revealed distinct chemical bond formations, while zeta potential measurements indirectly indicated compositional variations at the solid–liquid interface. Katende [14] demonstrated that oil-wet conditions result from organic adsorption, which is significantly influenced by factors such as pH, salinity, and crude oil properties. Buckley et al. [15] proposed four potential mechanisms for induced organic adsorption in carbonates: polar interactions, surface precipitation, acid–base interactions, and ionic bonding. Through these combined mechanisms, surfactants migrate from the oil–water interface to adsorb onto rock surfaces, forming hydrophobic monolayers that impart oil wettability to carbonate formations. Standnes [16] proposed a mechanistic model for wettability alteration mediated by anionic surfactants, showing that surfactant molecules adsorb onto organic layers via hydrophobic interactions, forming bilayer structures at rock interfaces. The reversibility of this process is due to the relatively weak hydrophobic bonding between surfactant molecules and hydrophobic surfaces. As a result, under oil-saturated conditions, pre-adsorbed surfactant molecules can be displaced by crude oil constituents through competitive adsorption.

Numerous laboratory experiments and field applications have shown that surfactants can effectively emulsify heavy oil, thereby enhancing oil recovery [17,18,19]. Injected chemicals form in situ emulsions with the crude oil in the reservoir, breaking the heavy oil into fine droplets. This process reduces capillary resistance to oil flow, alters rock wettability, and enhances the deformability and mobility of crude oil, which increases the sweep efficiency of the displacement system, ultimately enhancing oil recovery. Kokal et al. [20] indicated that reservoir pressure and temperature promote oil emulsification, while crude oil fractions strongly correlate with emulsification performance. The physical properties of crude oil play a fundamental role in determining the characteristics of the resulting emulsions. Chen et al. [21] found that the apparent viscosity of *O*/*W* emulsions in porous media is influenced by the ratio of droplet size to pore throat diameter. When the droplet size is smaller than the diameter of the pore throat, the apparent viscosity is nearly the same as that observed in stirred emulsification tests, as the Jamin effect is not present. In contrast, larger droplets encounter additional resistance due to the Jamin effect when passing through pore throats. Through visualized sand-pack flooding experiments, Lu et al. [22] directly demonstrated that the primary mechanism of chemical flooding with ultralow interfacial tension is the formation of oil banks, driven by the mobilization of residual oil via emulsions, followed by the displacement of the emulsion by the flooding fluid.

Understanding the mechanisms underlying surfactant adsorption-induced wettability alteration and heavy oil emulsion flow in porous media is essential for optimizing chemical flooding processes, especially surfactant-based EOR techniques [23]. In this study, the surface tensions of four structurally distinct surfactants and their interfacial tensions with heavy oil were measured, leading to the selection of BS-12 as the most effective emulsifier for Gudao heavy oil recovery. The interfacial adsorption mechanism of BS-12 on aged core samples, as well as the associated wettability alteration, was investigated using FTIR spectroscopy, contact angle measurements, and scanning electron microscopy (SEM). Additionally, core flooding experiments were performed to simulate thein situ formation of low-viscosity oil-in-water (*O*/*W*) emulsions under reservoir conditions. These experiments aim to provide theoretical insights into the screening and implementation of effective wettability alteration systems for oilfield applications.

## 2. Results and Discussion

### 2.1. FT-IR Characterization Results

**Figure 1:**
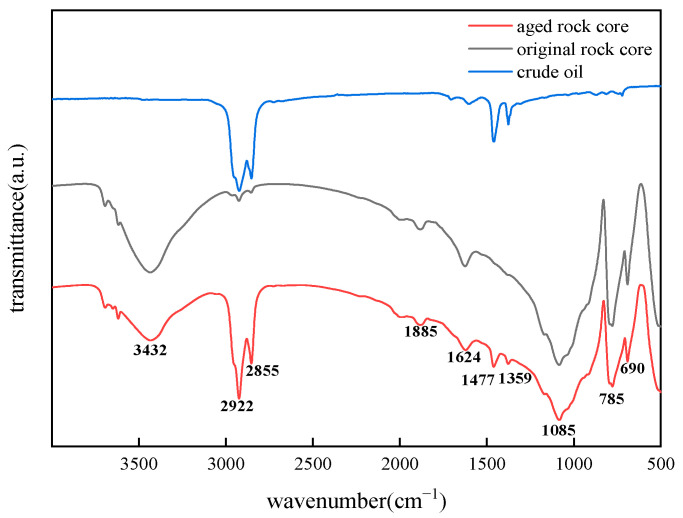
IR spectrum of crude oil and rock cores before and after aging.

**Figure 2:**
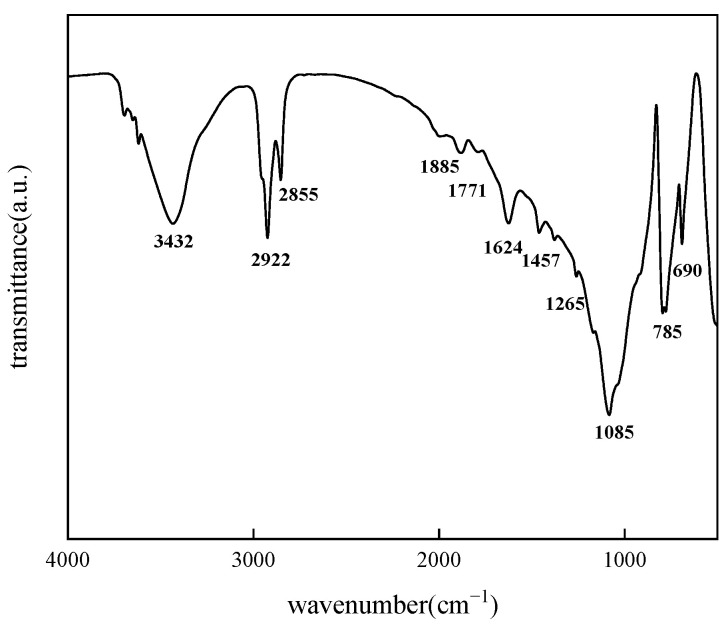
IR spectrum of aged rock cores soaked in BS-12.

The wettability alteration mechanism of oil-wet core surfaces and the adsorption behavior of BS-12 on these surfaces were investigated using FT-IR, as shown in Figure 1 and Figure 2. The spectrum of the aged oil-wet cores reveals characteristic peaks at 690 and 785 cm^−1^ (Si-O bending and stretching), 1085 cm^−1^ (asymmetric Si-O stretching), and 3432 cm^−1^ (O-H stretching), which align with the spectrum of the original rock core. Additionally, new absorption peaks are observed at 2855 and 2922 cm^−1^ (C-H stretching of alkyl chains -CH_3_ and -CH_2_) and 1359 cm^−1^ (C=O stretching), corresponding to those seen in the crude oil spectrum. These findings confirm the adsorption of oil components on the rock surfaces.

As illustrated in Figure 2, treatment with BS-12 led to a marked reduction in the absorption peaks at 2922 and 2985 cm^−1^ in the aged core samples, alongside the emergence of new characteristic peaks at 1265 and 1771 cm^−1^, corresponding to the stretching vibrations of C–N and C=O bonds, respectively. These spectral variations indicate the successful adsorption of surfactant molecules onto the solid surface, attributed to hydrophobic interactions between the hydrophobic chains of BS-12 and the hydrophobic groups of crude oil previously adsorbed on the aged core surface [24].

### 2.2. Surface (Interface) Properties of Surfactants

**Figure 3:**
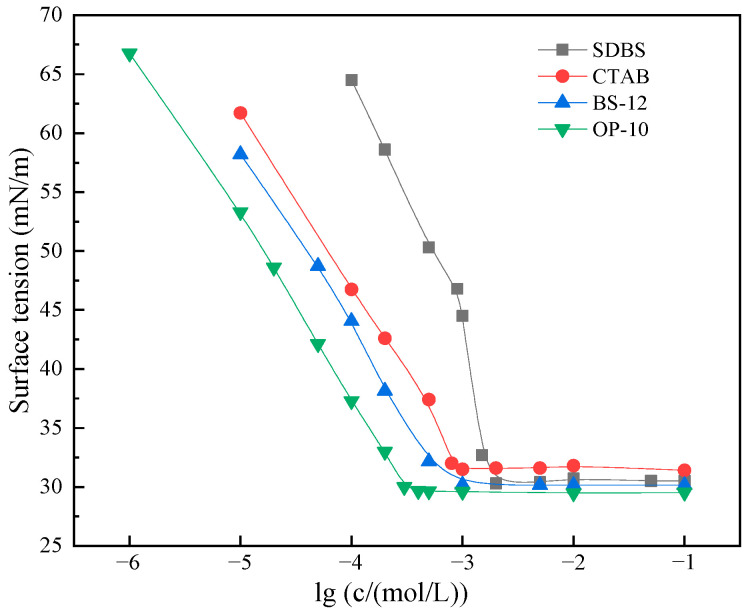
Surface tension curves of different surfactant solutions at 25 °C.

Figure 3 depicts the relationship between surface tension (γ) and the logarithm of concentration (lg *c*) for the emulsifier system. At low concentrations, a distinct negative linear correlation between surface tension and lg *c* was observed, suggesting the predominant adsorption of surfactant molecules at the air–liquid interface. When the surface tension reaches a plateau upon further increasing surfactant concentration, it can be conclusively determined that the solution has attained the critical micelle concentration (CMC). At this point, a dynamic chemical potential equilibrium is established between surfactant monomers in the bulk solution and the micellar phase (*µ_monomer_ = µ_micelle_*), resulting in the coexistence of both monomeric and micellar phases in the system: the excess monomers self-assemble into micelles through the inward association of hydrophobic tails and outward orientation of hydrophilic headgroups. The relevant parameters of the surfactant are listed in Table 1 (the calculation method refers to Equations (1)–(3)).

The surface tension at the CMC, denoted as *γ_CMC_*, is a crucial parameter for evaluating a surfactant’s ability to reduce surface tension. The adsorption efficiency of surfactants can be quantified using the *pC_20_* value, which is defined as the negative logarithm of the concentration needed to lower surface tension by 20 mN·m^−1^ (i.e., *pC_20_* = −log *C_20_*). A higher *pC_20_* value reflects a stronger adsorption capacity at the interface, implying that lower surfactant concentrations are required to achieve the same reduction in interfacial energy. Although all four surfactants listed in Table 1 demonstrated excellent surface tension reduction capabilities, BS-12 emerged as the most effective surfactant, due to its optimal combination of key parameters: a low CMC (6.26 × 10^−4^ mol·dm^−3^), favorable *γ_CMC_* (30.15 mN·m^−1^), high surface packing (*Γ_max_* = 1.42 μmol·m^−2^), and elevated surface activity (*pC_20_* = 4.54). A low CMC and robust interfacial activity significantly enhance the techno-economic efficiency of chemical flooding in heavy oil recovery.

**Figure 4:**
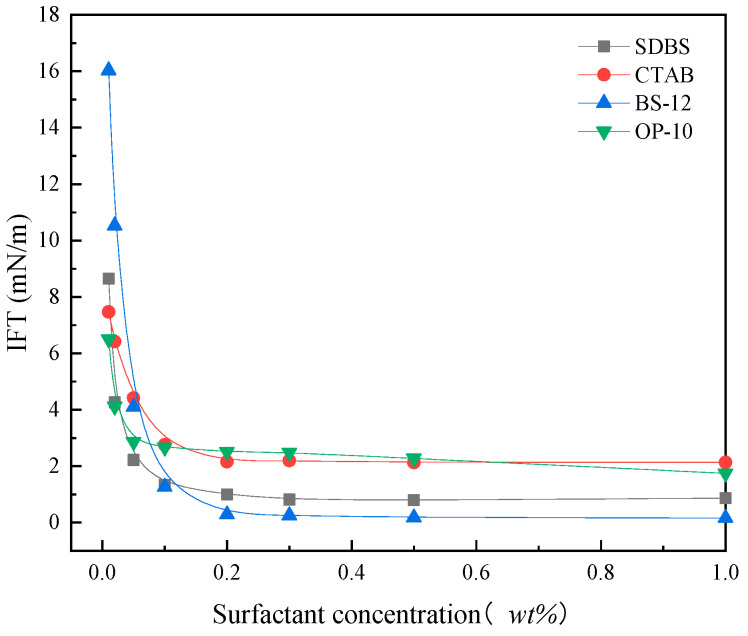
Variation curve of interfacial tension of surfactant solution with concentration at 40 °C.

Figure 4 presents the interfacial tension (IFT) variation between crude oil and four emulsifiers as a function of concentration. The IFT decreases with increasing emulsifier concentration, reaching a plateau beyond a certain threshold. Both the anionic surfactant SDBS and the zwitterionic surfactant BS-12 reduce the IFT to the order of 10^−1^ mN·m^−1^. The study of Sun et al. [25] indicated that betaine had generally high interfacial film strength with crude oil, at this time, the slight difference in film strength had a negligible effect on enhancing recovery. The main contributing factor was the difference in interfacial tension between the oil and water. Ultralow IFT could increase the stripping efficiency of oil in the pores and reduce the energy required for emulsification or the initiation of oil droplets. Betaine-type surfactants [26] synergize with crude oil’s native active components to reduce IFT to ultralow levels. The optimal spatial arrangement of hydrophobic chains facilitates co-adsorption with crude oil surfactants at the interface, forming densely packed interfacial films that trigger abrupt declines in dynamic IFT measurements.

**Figure 5:**
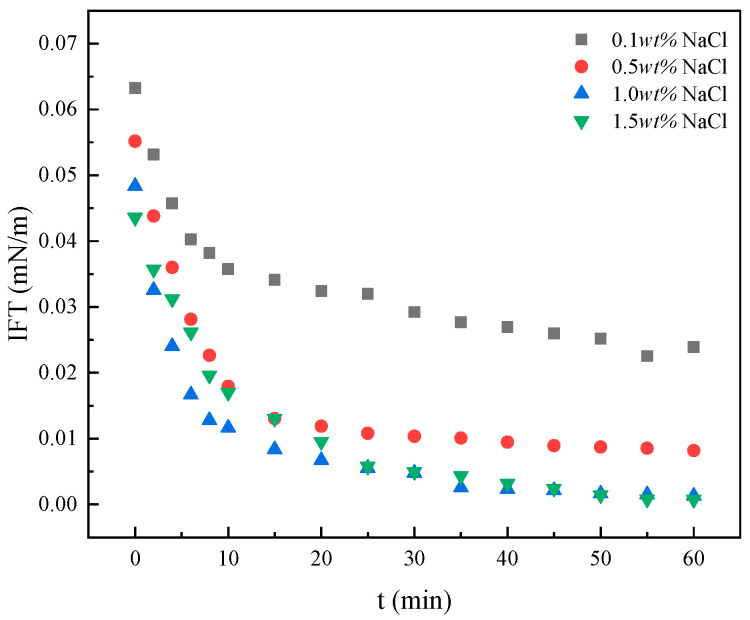
IFT of BS-12 at oil–water interface under different salt contents at 40 °C.

Notably, BS-12 exhibits enhanced interfacial activity under saline conditions (Figure 5), achieving an ultralow IFT of 0.001135 mN·m^−1^ (on the order of 10^−3^ mN·m^−1^) in a 1 wt% NaCl solution. A study on the synergistic effects of betaine molecular structures and formation brine composition on IFT reduction further elucidated the cooperative mechanisms of salts in lowering IFT [27]. The addition of NaCl screens electrostatic repulsion among charged surfactant molecules. Increased ionic strength introduces counterions (Na^+^ or Cl^−^) that compress the electric double layer, thereby weakening repulsive interactions between polar headgroups and promoting tighter interfacial molecular packing. As a result, micelle size decreases, and the synergistic effect of salt and surfactant enhances hydrophobic tail interactions due to the closer arrangement of polar heads. This leads to increased surfactant adsorption at the interface, where the densely packed molecules suppress the asymmetric interactions of water molecules, lowering the interfacial energy and thereby reducing IFT [28]. Considering the inherent salinity of formation water in reservoirs, the ability of BS-12 to sustain ultralow IFT at elevated NaCl concentrations highlights its suitability for salinity-tolerant enhanced oil recovery applications.

### 2.3. Wettability Performance of Surfactants

**Figure 6:**
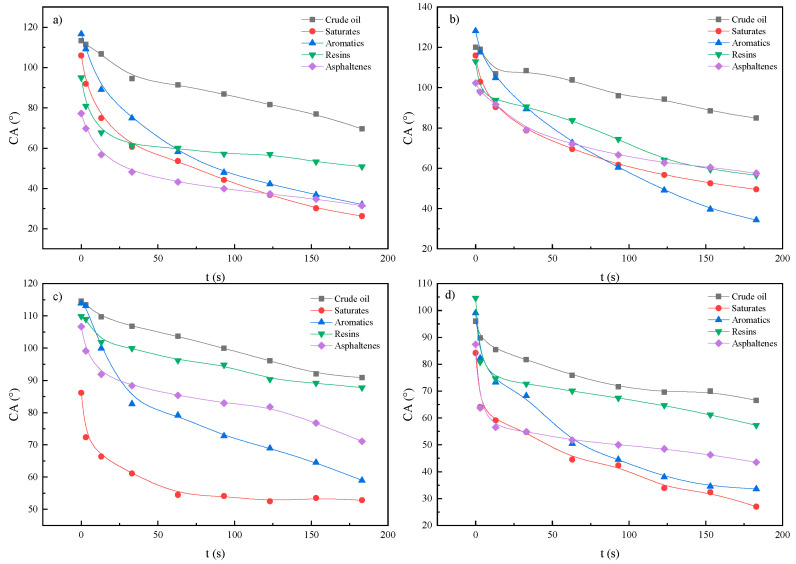
Contact angle variation curve of surfactant solution (0.05 wt%) on surface of rock cores adsorbing different crude oils and their components: (**a**) SDB; (**b**) CTAB; (**c**) BS-12; (**d**) OP-10.

Heavy oil is a complex mixture consisting of multiple polar and non-polar chemical fractions, including asphaltenes, resins, saturates, and aromatics. These fractions exhibit distinct molecular structures and varying distributions within crude oil, which result in different adsorption behaviors on rock surfaces. These behaviors, in turn, affect the wettability of the rock to varying extents. Changes in contact angle can serve as an indirect indicator of wettability alterations on oil-wet core surfaces. The heavy oil fractions were separated by column chromatography, and each fraction was used to treat the core samples individually through aging. Wettability measurements were then performed. The apparent wettability results for rock surfaces adsorbed with different heavy oil fractions are presented in Figure 6.

**Figure 7:**
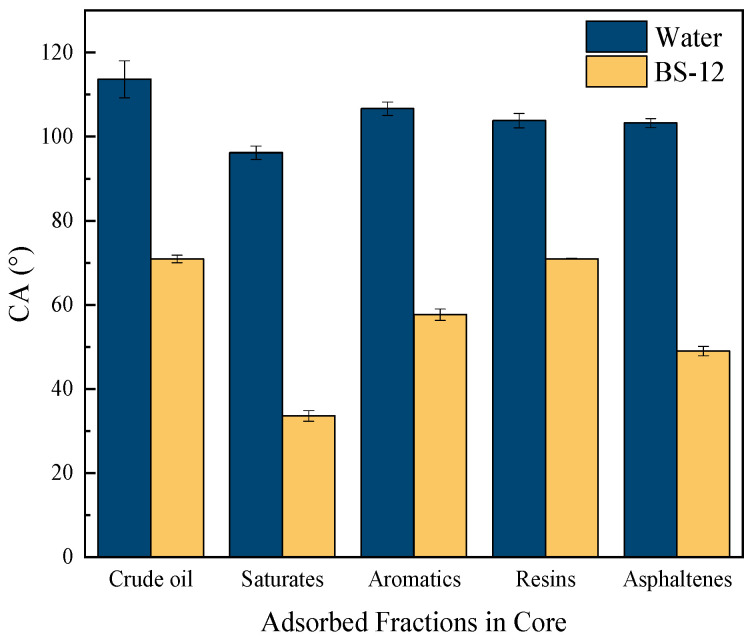
Contact angles of water and BS-12 solution on surface of rock cores adsorbing different crude oil and their fractions.

As illustrated in Figure 6, the contact angles of all surfactants decreased markedly with increasing contact time, demonstrating rapid spreading behavior followed by stabilization after approximately 2 min. This behavior suggests that the wetting process involves two distinct stages: a rapid adsorption phase of the surfactant followed by the establishment of equilibrium. This phenomenon is attributed to the dynamic adsorption of surfactant molecules at the solid–liquid interface, where molecular rearrangement leads to the formation of a bilayer adsorption structure. The hydrophobic tails interact with crude oil fractions, while the hydrophilic headgroups orient outward, resulting in a gradual reduction in contact angles. This interfacial reorganization also modifies other surface properties, including surface charge characteristics, microscopic adsorption morphology, and the distribution of chemical functional groups [29]. Figure 7 indicates that, compared to pure water, the BS-12 solution significantly enhances the surface hydrophilicity of cores adsorbed with various fractions, with a particularly pronounced wettability reversal observed on aromatic and asphaltene-coated surfaces (which are initially strongly oil-wet). BS-12 exhibits rapid spreading on oil-wet core surfaces, facilitating its effective action along the pore channel surfaces of formation cores, thereby significantly improving the unblocking performance of the surfactant system.

The modification of rock wettability is primarily influenced by three fundamental intermolecular interactions: electrostatic forces, hydrophobic effects, and specific attractive interactions between the polar components of crude oil and surfactant molecules. These interactions collectively govern the efficiency of wettability alteration processes [4]. Hou et al. [30] demonstrated that the ionic pairing mechanism plays a crucial role in wettability modification by the cationic surfactant CTAB, where electrostatic attraction occurs between the positively charged headgroups of CTAB and the carboxylate groups in crude oil. Standnes et al. [16] proposed an adsorption-based mechanism for anionic surfactants, in which wettability alteration results from hydrophobic interactions between surfactant tails and oil-wet surfaces. Their study highlighted that surfactant adsorption can effectively alter the wettability of oil-wet solid surfaces, converting them to water-wet states through these molecular-scale processes.

At the molecular scale, surfactant adsorption describes the migration of surfactant molecules from the bulk solution phase to surfaces or interfaces. Different surfactants display distinct adsorption configurations on rock surfaces, resulting in varying degrees of wettability alteration. The extent of contact angle variation is influenced by multiple factors.

**Figure 8:**
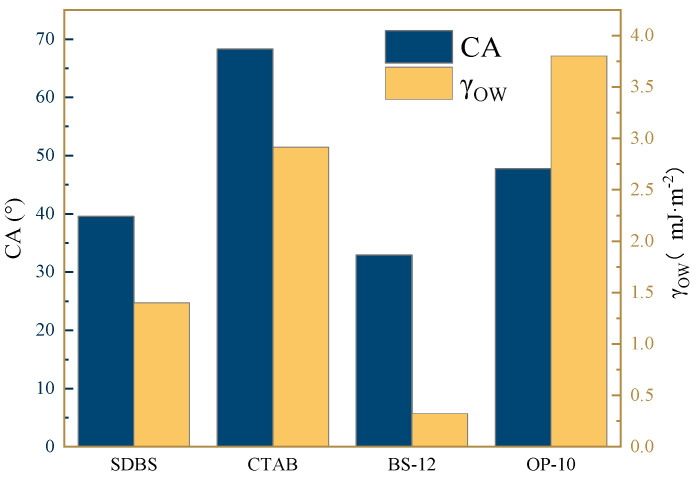
Wettability and corresponding adhesion work of three-phase system under different surfactant conditions.

The oil–solid adhesion work (*W_OS_*) was calculated based on Equation (4) by integrating interfacial tension data with contact angle measurements. This adhesion work represents the thermodynamic work necessary to separate two contacting phases [31] and serves as an indicator of the difficulty in detaching crude oil from solid surfaces [32]. Figure 8 presents the comparative results of contact angles and adhesion work for aged cores treated with four distinct surfactant systems. The results clearly show that the zwitterionic surfactant BS-12 induces the most significant wettability alteration on oil-wet aged core surfaces, demonstrating superior surface-modifying performance compared to the other surfactants tested.

### 2.4. SEM Images Results

**Figure 9:**
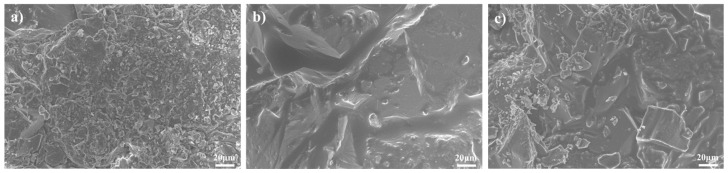
SEM images of core samples under different treatment conditions: (**a**) untreated original core, (**b**) crude oil-adsorbed aged core, (**c**) BS-12-treated core.

To investigate the interfacial interactions between emulsifier solutions, rock substrates, and crude oil, SEM was utilized to compare three different core surface conditions: untreated original cores, aged cores with crude oil adsorption, and emulsifier-treated cores. The representative microstructural characteristics are shown in Figure 9.

The results show that the surface of the crude oil-adsorbed core (Figure 9b) presents a relatively smooth morphology due to the oil coverage, with some regions exhibiting particularly thick oil adsorption that fully conceals the pore structures. In contrast, the emulsifier-treated core (Figure 9c) demonstrates significant surface reconstruction, where partial oil removal exposes reopened pore networks, which can facilitate oil migration. The continuous hydrophobic layer formed by crude oil adsorption is partially detached, revealing the underlying microstructure of the native core. This observed microstructural change, in conjunction with macroscopic wettability measurements, is attributed to the dual mechanism of the emulsifier: its hydrophobic tails penetrate and disrupt the oil–rock adhesion, while its zwitterionic headgroups form hydrophilic domains on the surface.

### 2.5. Core Displacement Experiment Results

**Figure 10:**
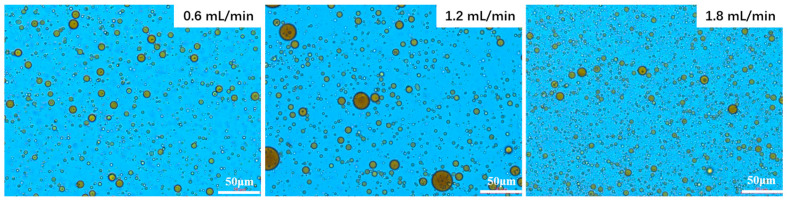
Microscopic images of emulsions under different velocities of flow.

**Figure 11:**
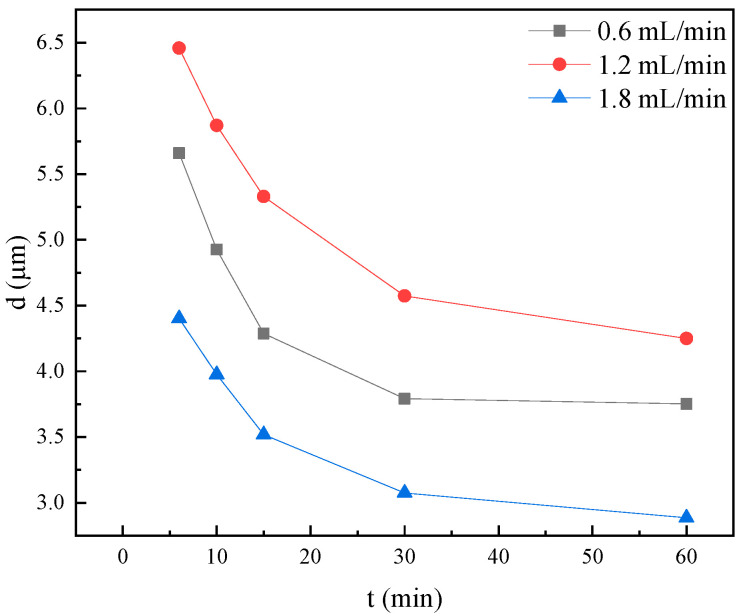
Particle size distribution of emulsions under different velocities of flow.

The migration of crude oil through reservoir porous media often occurs in the form of emulsions, the characteristics of which are determined by their interfacial properties. These emulsion characteristics, such as droplet size distribution, emulsified oil content, and stability, all of which are indicators of interfacial behavior, play a crucial role in influencing seepage dynamics and ultimate recovery efficiency. Importantly, the compatibility between the size of emulsion droplets and the diameter of the reservoir pore throats directly affects the mobility of crude oil. A comprehensive study of the interactions between emulsion characteristics and seepage behavior forms the theoretical basis for optimizing emulsion flooding techniques, thus enhancing oil recovery performance. Figure 10 shows the microscopic images of emulsions under different flow rates, while Figure 11 presents the corresponding particle size distribution.

As shown in Figure 11, the emulsion droplet size significantly decreased within the first 30 min of outflow, after which the size distribution gradually stabilized, reaching a steady state with minimal variation by 60 min. Droplet size reduction occurred at flow rates both below and above 1.2 mL/min, with a more pronounced decrease at higher flow rates, as clearly illustrated in the corresponding microscopic images (Figure 10). This variation in size is likely due to changes in interfacial wettability and macrochannel plugging [33]. The effectiveness of surfactant-induced wettability alteration is strongly time-dependent, as demonstrated by the evolution of the contact angle: a rapid initial decrease followed by an asymptotic approach to equilibrium. Low-flow conditions provide sufficient time for both complete wettability reversal and enhancement in interfacial activity through surfactant rearrangement, consistent with the findings of Ahmadi-Falavarjan et al. [34].

At lower flow velocities, the extended contact time between oil and water within the pipeline facilitates a more thorough interaction, allowing for a longer reaction duration between heavy oil and the emulsifier, which results in the formation of emulsion droplets with smaller diameters. However, the insufficient hydrodynamic driving force under these conditions fails to mobilize the residual oil clusters left behind after water flooding, thereby limiting both the volume of emulsion produced through fragmentation mechanisms and the overall rate of emulsion generation. In this flow regime, most emulsion droplets are produced via shear-induced mechanisms, leading to typically smaller droplet sizes [16]. Although lower injection velocities initially result in relatively larger droplets, the reduced hydrodynamic forces cause these larger droplets to be preferentially retained within the porous matrix due to the diminished impact energy, compared to high-flow-rate conditions. In contrast, higher flow rates promote the channeling of the emulsifier solution through high-permeability zones while subjecting the oil phases to intensified mechanical shear, which induces droplet elongation, deformation, and eventual rupture [35], thereby producing emulsions with smaller characteristic diameters through enhanced dispersive action.

**Figure 12:**
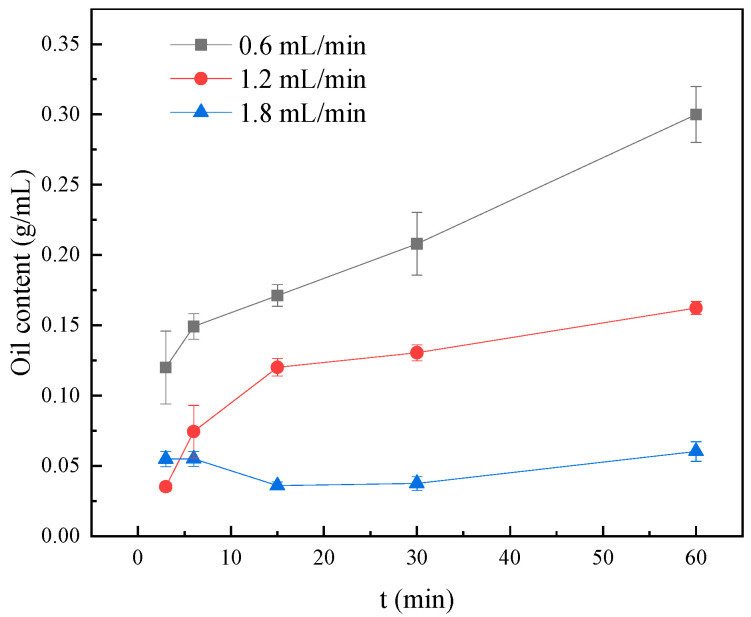
Oil content of emulsions under different velocities of flow.

Flow velocity significantly affects the emulsified oil content within porous media, with higher flow rates leading to a reduction in oil emulsification. As shown in Figure 12, at a flow rate of 1.8 mL/min, the oil content remained below 0.06 g/mL throughout the entire experiment. In contrast, at 1.2 mL/min, the oil content gradually increased over time, reaching 0.16 g/mL after 60 min. The highest oil content was observed at the low flow rate of 0.6 mL/min, which can be attributed to the slower expansion of fluid sweep efficiency under reduced flow conditions. Furthermore, the restricted mobility of oil droplets through narrow pore throats promotes phase separation, where the aqueous phase preferentially flows through narrow channels with longer residence times, resulting in increased water retention within the porous matrix. Consequently, the produced fluid exhibits a lower water cut and a corresponding increase in oil content [36].

## 3. Experimental Section

### 3.1. Reagents and Instruments

The chemical reagents used in this study include dodecyl betaine (BS-12), cetyltrimethylammonium bromide (CTAB, AR), and octylphenol ethoxylate-10 (OP-10, AR), all purchased from Macklin Biochemical Technology Co., Ltd. (Shanghai, China). Sodium dodecylbenzenesulfonate (SDBS, AR), sodium chloride (NaCl, AR), and toluene (AR) were obtained from Sinopharm Chemical Reagent Co., Ltd. (Shanghai, China). The crude oil sample used was Gudao heavy oil, with a density of 0.9282 g/mL and a viscosity of 965 mPa·s at 50 °C. Purified water (double-distilled deionized water) for all experiments was prepared in the laboratory. Test instruments included an automatic surface tensiometer (Model JK99B, Shanghai Zhongchen Digital Technology Equipment Co., Shanghai, China), an interfacial tensiometer (Model CNG700, Beijing Shengwei Jiye Technology Co., Ltd., Beijing, China), a contact angle analyzer (Model DSA25, Krüss GmbH, Hamburg, Germany), a Fourier-transform infrared spectrometer (Model Frontier, PerkinElmer Inc., Shanghai, China), a scanning electron microscope (Model Sigma 500, Carl Zeiss AG, Oberkochen, Germany), and a polarized light microscope (Model BX51, Olympus Corporation, Tokyo, Japan).

### 3.2. Experimental Procedures

#### 3.2.1. Core Surface Preparation

Crude oil was initially separated into four fractions—saturates, aromatics, resins, and asphaltenes—using column chromatography. Core slices were then immersed in crude oil and the four fractions and aged at a constant temperature of 65 °C for 24 h to produce aged core surfaces that adsorbed different fractions. After aging, the core samples were subjected to oscillation in a surfactant solution at 65 °C for an additional 24 h. Finally, the treated cores were vacuum-dried at 40 °C.

#### 3.2.2. Fourier-Transform Infrared Spectroscopy (FT-IR) Analysis

The core powder samples were analyzed by FT-IR, and the spectral data obtained were processed to produce infrared spectra for both the aged core powder and the core powder treated with emulsifier.

#### 3.2.3. Wettability Measurement

**Figure 13:**
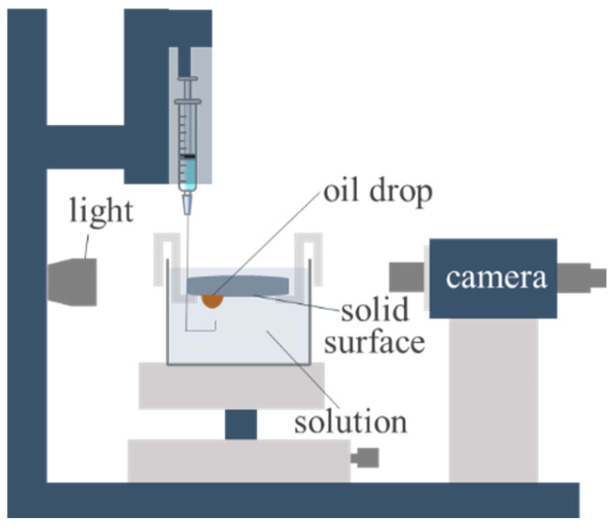
Schematic diagram of measuring contact angles using reverse sessile drop method.

The wettability of the prepared core surfaces was characterized using a contact angle goniometer. Static contact angles were determined at three randomly selected points on each core surface by the sessile drop method, employing 2.5 μL liquid droplets. Considering the lower density of oil relative to aqueous solutions, the inverted pendant drop technique was used to determine the oil–water–solid three-phase contact angles, as depicted in Figure 13.

#### 3.2.4. Surface Tension Measurement

Surface tension measurements of surfactant solutions at varying concentrations were conducted using the Wilhelmy plate method. A γ/lg *c* curve was constructed to identify the critical micelle concentration (CMC). In addition, the Gibbs adsorption equation was utilized to determine the maximum surface adsorption (*Γ_max_*), the minimum molecular cross-sectional area (*Amin*), and the surface adsorption efficiency (*pC_20_*) [37,38,39].

(1) $$\Gamma_{\max}=-\frac{1}{2.303n\mathrm{RT}}\times {\left(\frac{d\gamma }{\mathrm{dlg}c}\right)}_{T}$$

(2) $$A_{\min}=\frac{1}{N_{A}\Gamma_{\max}}$$

(3) $${\mathrm{pC}}_{20}=\frac{\gamma_{0}{-20-\gamma }_{\mathrm{cmc}}}{{2.303n\mathrm{RT}\Gamma }_{\max}}-\mathrm{lg}\;\mathrm{cmc}$$

where *γ*—surface tension, mN·m^−1^; *c*—surfactant concentration, mol·L^−1^; *T*—298.15 K; *R*—8.314 J·mol^−1^·K^−1^; *γ*_0_—71.8 mN·m^−1^; *N_A_*—6.022 × 10^23^ mol^−1^.

#### 3.2.5. Interfacial Tension (IFT) Measurement

IFT between the emulsifier solutions and crude oil was measured using the spinning drop method. Prior to measurement, the surfactant solutions (0.20 wt%) were equilibrated in the instrument at 40 °C for 20 min. The measurements were performed using an interfacial tensiometer, with each sample tested in triplicate and the results averaged.

Adhesion work (*W_OS_*) can be calculated based on Young’s equation by incorporating the oil–water interfacial tension (*γ_OW_*) and the contact angle (*θ*) in the oil–water–solid three-phase system [31]:

(4) $$W_{\mathrm{OS}}=\gamma_{\mathrm{OS}}\left(1+\cos\theta \right)$$

#### 3.2.6. Scanning Electron Microscopy (SEM) Analysis

The core samples were characterized using SEM to analyze their surface morphology. Cubic specimens (1 cm × 1 cm) were placed on the sample stage and sputter-coated with gold prior to imaging. SEM micrographs were captured at optimized focus and magnification.

#### 3.2.7. Core Flooding Experiment

**Figure 14:**
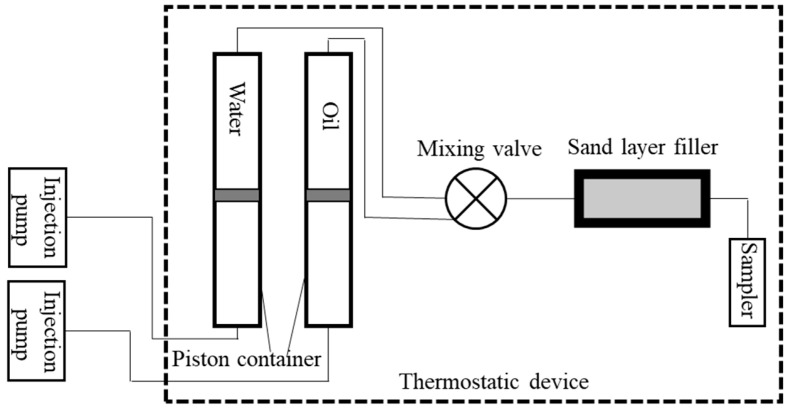
Diagram of displacement experiment device.

The core flooding experimental setup was constructed as shown in Figure 14. Approximately 80 g of Gudao heavy oil was loaded into the oil tank, while the displacing fluid (0.2 wt% BS-12 solution) was introduced into the water tank. The pump was calibrated to maintain consistent flow rates (0.6~1.8 mL/min) and oil-to-water ratios (1:5) through the core holder. The experiment was conducted at a controlled temperature of 40 °C. Effluent emulsions were collected at various time intervals for subsequent analysis, including oil content determination. A microscopic examination was conducted to observe and record the size and morphology of the emulsion droplets. The microscopic images were then quantitatively analyzed using the publicly available software Image J (version 1.8.0) to determine the droplet size distribution of the emulsions.

## 4. Conclusions

A comparative analysis of interfacial properties across four surfactant types revealed that the zwitterionic surfactant BS-12 exhibits outstanding performance, with a *γ_CMC_* of 30.15 mN·m^−1^, a *pC_20_* of 4.54, and a reduction in interfacial tension to 10^−1^ mN·m^−1^. Notably, in a 1.0 wt% NaCl solution, BS-12 achieved an ultralow interfacial tension of 0.001135 mN·m^−1^ (on the order of 10^−3^ mN·m^−1^), demonstrating its superior suitability for Gudao heavy oil exploitation. FT-IR, SEM, and contact angle measurements collectively revealed the microscopic mechanisms underlying the remarkable ability of BS-12 to alter wettability. The surfactant molecules form bilayer adsorption structures through hydrophobic interactions between their alkyl chains and the crude oil components adsorbed on aged quartz surfaces. BS-12 spreads rapidly on oil-wet core surfaces, causing an immediate reduction in the contact angle and efficiently reversing wettability from oil-wet to water-wet. The low adhesion work facilitates the effective performance of the surfactant within reservoir pore networks, thereby enhancing the system’s ability to remove blockages. The emulsification process within porous media occurs continuously, with low-flow-rate conditions (0.6 mL/min) promoting prolonged interactions between oil and the emulsifier. This extended contact time enhances oil–water interfacial reactions in pore spaces, induces wettability reversal (from oil-wet to water-wet), improves interfacial activity, and ultimately leads to superior oil recovery performance.

This study investigates the optimal conditions for surfactant-mediated wettability regulation and the mechanisms underlying wettability reversal. BS-12 significantly enhances crude oil detachment from pore surfaces by achieving ultralow interfacial tension and inducing wettability alteration, thereby improving the contact efficiency of displacing fluids and oil recovery capability. The continuous emulsification process of *O*/*W* emulsions in porous media is also governed by interfacial adsorption dynamics and wettability modulation. These findings provide an efficient technical pathway for chemical flooding strategies in Gudao heavy oil reservoirs and offer potential solutions for the development of unconventional hydrocarbon resources, demonstrating significant effects for enhancing oil recovery through regulating interface properties.

## Figures and Tables

**Table 1:** Physical and chemical parameters of different surfactants.

Surfactant	*M*/g·mol^−1^	CMC/mol·dm^−3^	*γ_CMC_*/mN·m^−1^	*Γ_max_*/μmol·m^−2^	*A_min_*/nm^2^	*pC_20_*
SDBS	348.5	1.92 × 10^−3^	30.50	2.23	0.74	3.55
CTAB	364.5	9.71 × 10^−4^	31.56	1.33	1.26	4.36
BS-12	335.5	6.26 × 10^−4^	30.15	1.42	1.17	4.54
OP-10	646	3.17 × 10^−4^	29.63	2.76	0.60	4.91

## Data Availability

The original contributions presented in this study are included in the article; further inquiries can be directed to the corresponding author.
